# A Fusion Algorithm of Object Detection and Tracking for Unmanned Surface Vehicles

**DOI:** 10.3389/fnbot.2022.808147

**Published:** 2022-04-27

**Authors:** Zhiguo Zhou, Xinxin Hu, Zeming Li, Zhao Jing, Chong Qu

**Affiliations:** ^1^School of Integrated Circuits and Electronics, Beijing Institute of Technology, Beijing, China; ^2^China State Shipbuilding Corporation Limited, Shanghai Marine Diesel Engine Research Institute, Shanghai, China

**Keywords:** unmanned surface vehicle (USV), deep learning, object detection, object tracking, fusion of detection and tracking

## Abstract

To provide reliable input for obstacle avoidance and decision-making, unmanned surface vehicles (USV) need to have the function of sensing the position of other USV targets in the process of cooperation and confrontation. Due to the small size of the target and the interference of the water and sky background, the current algorithms are prone to missed detection and drift problems when detecting and tracking USV. Therefore, in this paper, we propose a fusion algorithm of detection and tracking for USV targets. To solve the problem of vague features in the single-frame image, high-resolution and deep semantic information are obtained through a cross-stage partial network, and the anchor and convolution structure in the network has been improved given the characteristics of USV; besides, to meet the real-time requirements, the detected target is quickly tracked through correlation filtering, and the correlation characteristics of multi-frame images are obtained; then, the correlation characteristics are used to significantly reduce missed detection, and the tracking drift problems are corrected, combined with high-resolution semantic features of a single frame. Finally, the fusion algorithm is designed. In this paper, we constructed a picture dataset and a video dataset to test the effect of detection, tracking, and fusion algorithm separately, which proves the superiority of the fusion algorithm in this paper. The results show that, compared with a single detection algorithm and tracking algorithm, the fusion one can increase the success rate by more than 10%.

## Introduction

As a new type of water surface equipment, unmanned surface vehicles (USV) can complete reconnaissance missions with lower costs and higher efficiency, which has important strategic significance in maritime games. In the process of cooperation and confrontation of USV, detection, and tracking algorithms are used to perceive the position of other USV and provide reliable input for autonomous path planning and decision-making. The USV is usually small in size, which makes it difficult to be found by the visual system with vague features. For object detection algorithms, too few features lead to missed detection; at the same time, affected by the background of the water surface (Fefilatyev et al., 2012), the tracking algorithm will inevitably learn the background noise, and the drift problem that exists, which affects the detection accuracy.

Object detection includes traditional image processing methods (Dalal and Triggs, 2005; Felzenszwalb et al., 2010) and deep learning methods. Traditional methods have higher requirements for the images to be recognized. They can perform better in the situation that the water surface is well lit and the target greatly differs from the background. However, the robustness is poor when applied to specific scenes. Deep learning algorithms generally include two-stage algorithms based on candidate boxes and one-stage algorithms based on regression. Two-stage algorithms for general object detection often perform high accuracy and low speed, such as Fast-RCNN (He et al., 2019), Faster-RCNN (Ren et al., 2015), Mask-RCNN (Zhang et al., 2020), etc.; one-stage algorithms, such as the You Only Look Once (YOLO) series (Cai et al., 2020), Single Shot Multibox Detector (SSD) (Liu et al., 2016), RetinaNet (Lin et al., 2017a), etc., usually perform faster detection speeds. Object detection algorithms designed for small targets include feature pyramid network (FPN) (Lin et al., 2017b), Scale Invariance in Object Detection (SNIP) (Singh and Davis, 2018), Cascade R-CNN (Cai and Vasconcelos, 2018), ALFNet (Liu et al., 2018), DetNet (Li et al., 2018), etc. At present, there are few results for water surface object detection, and most of them use traditional algorithms, and the robustness is relatively poor.

The object detection algorithm generally only analyzes the spatial features in a single frame without using the relationship between the video frames, which is also at a low speed. The object tracking algorithms are based on correlation filtering (Tang et al., 2020), which firstly extract image features and then calculate the relevance between the current frame and the previous one. Bolme et al. (2010) proposed the MOSSE algorithm, which selects the minimum mean square error-index to calculate the characteristic response map to quickly obtain the target pixel coordinates. Henriques et al. (2012) designed CSK, the KCF (Henriques et al., 2015) algorithm, a circulant matrix was introduced to enhance the data samples; thus, the accuracy of tracking can be improved, and gradient histogram features are adapted to describe the foreground and background; furthermore, the HOG feature is used to describe the foreground object and the background in the KCF algorithm, and the Gaussian kernel function is introduced to achieve a better tracking effect and real-time performance at the same time. The feature extraction process of these algorithms is often relatively simple, so they are likely to be affected by the drift problems and other phenomena during the tracking process. With the fast development of deep learning algorithms, object tracking algorithms based on convolutional neural networks perform better in accuracy (Zhang et al., 2017). Chen et al. (2020) proposed MDNet, which uses a multi-domain convolutional neural network for tracking tasks. Nam and Han (2016) approached the tracking problem using a correlation filter with a convolutional feature. Danelljan et al. (2015) used RNN and the attention model to get better performance. However, neural networks would cost a lot of computing resources, so, they are slow.

To solve the problem of missed detection and the drift problem in the detection and tracking of USV on the water surface, we propose a fusion algorithm of detection and tracking for USV, which applies a deep learning object detection algorithm to extract the precise position information of USV in a single frame. In the video sequence, the correlation filter tracking algorithm is used to obtain the correlation characteristics between video frames to reduce the probability of missed detection. Finally, the output of detection and tracking is merged to realize the complementation of the two algorithms.

## Materials and Methods

**Figure 1:**
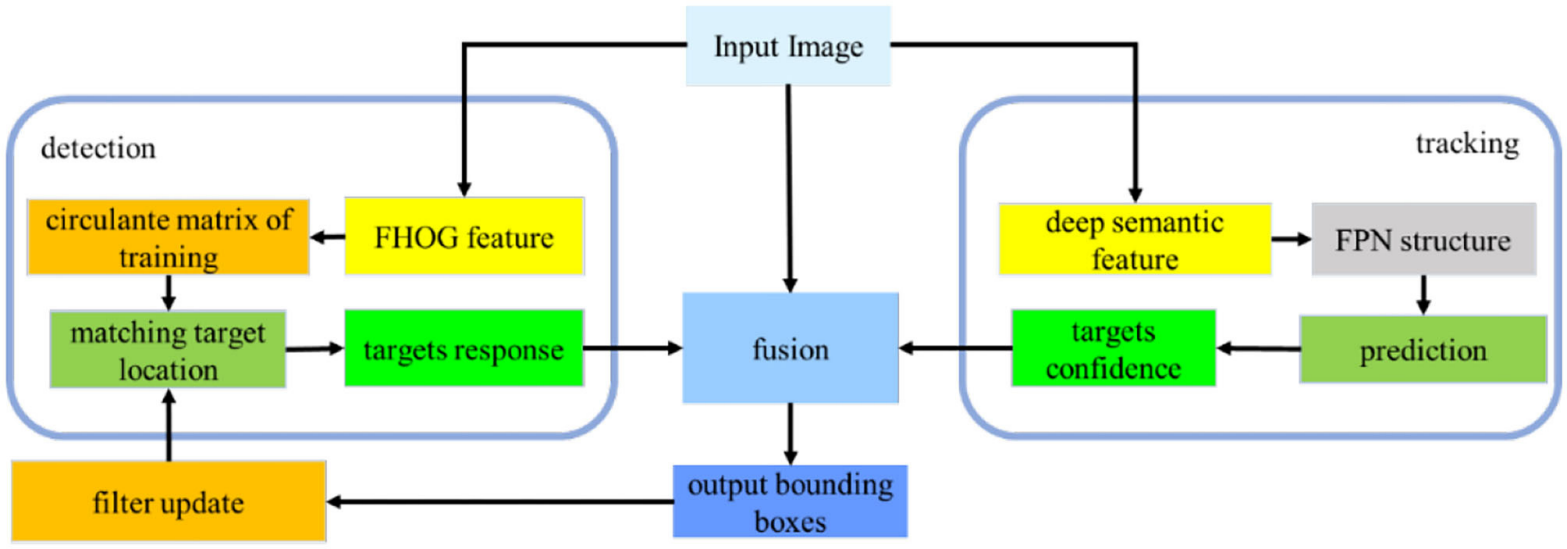
An algorithm framework diagram.

The fusion algorithm of detection and tracking is designed for the USV targeting, which includes deep learning detection on a single frame of the image, correlation filter tracking on video sequence, and a fusion module. At first, we detected the objects in the video image through the YOLOv5 algorithm, and then we used the KCF algorithm to continuously track the surface target. After tracking a certain number of frames, the detection mechanism is introduced again, and then the output of the target frame is completed through the feature fusion algorithm based on machine learning; if new targets appear in the field of view, then the new ones are initialized and tracked. The algorithm framework is shown in Figure 1.

### Object Detection for USV

In this paper, we adopted the framework based on the YOLOv5 (you only look once) algorithm to realize the feature extraction and detection of the targets in a single frame.

**Figure 2:**
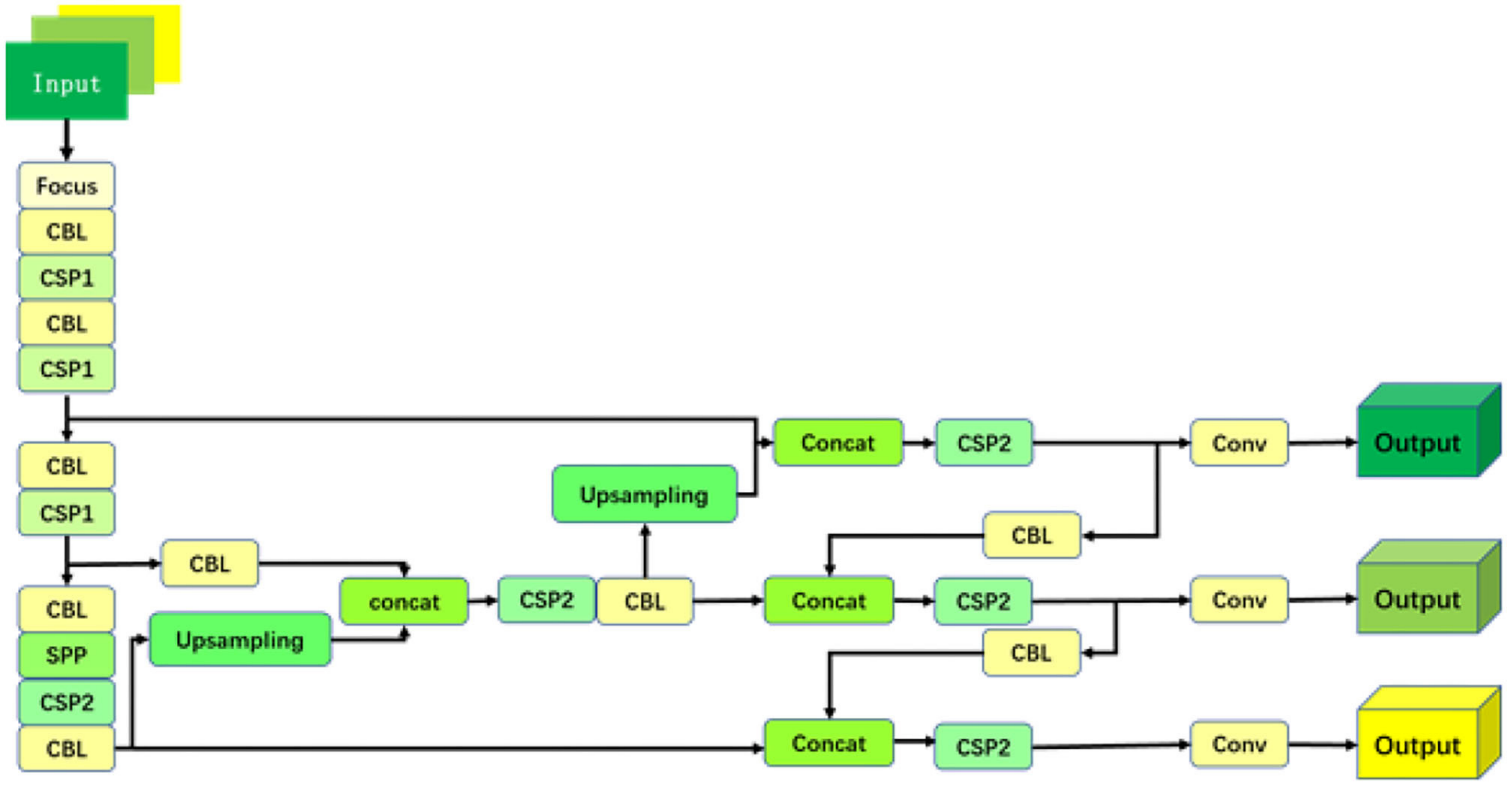
A detection algorithm network structure diagram.

At the input part, firstly, the Mosaic data enhancement method is applied to improve the algorithm effect at the sample level, and then, the centralized convolution (Focus) operation is applied to convert the original input image (with the size of 608 × 608 × 3) into a feature map (with the size of 304 × 304 × 12) through a slicing operation. After the convolution operation, the feature map is finally generated with the size of 304 × 304 × 32. The cross-stage partial (CSP) module is used to divide the feature map of the base layer into two branches, which reduces the amount of calculation and reduces the possibility of gradient disappearance. The network contains two cross-stage structures, which are referred to as cross-stage partial 1 (CSP1) and cross-stage partial 2 (CSP2). The feature pyramid network (FPN) structure (Chen et al., 2020) is also introduced to improve the detection accuracy of small objects. It is realized by the following method. First, the top-level features are up-sampled and merged with the lower-level feature maps, and then, high-order semantic features and shallow features with high-resolution are merged to complete the process. After the FPN layer, a PAN structure that conveys strong position features from the bottom up is added to further improve the feature-extracting ability. Finally, three different scales of the feature maps are exported, which will be used for prediction. The network structure is shown in Figure 2, where the convolution module represents the cascade of convolutional layers, batch normalization, and activation functions.

**Figure 3:**
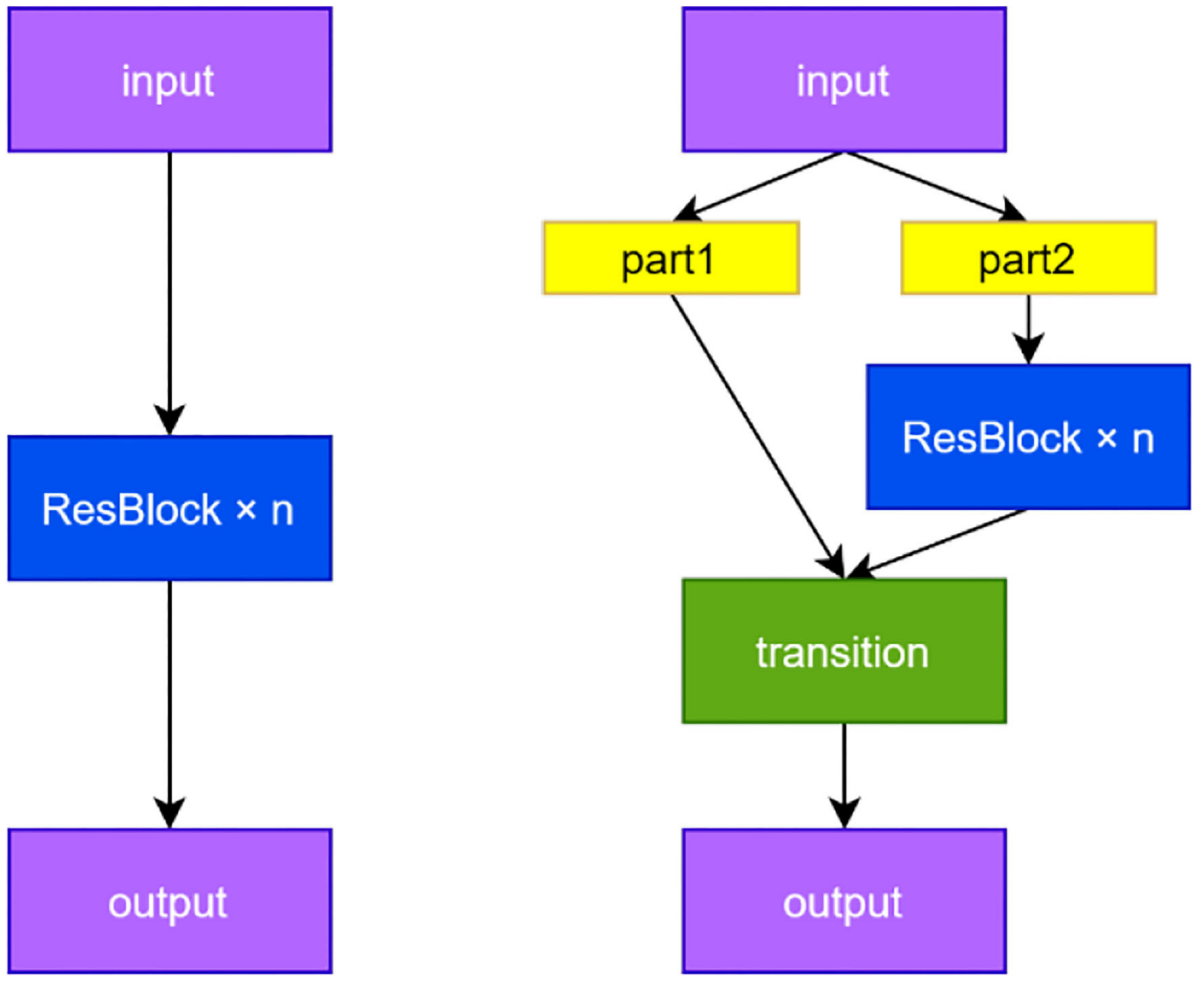
ResNet and CSPResNet structure.

The YOLOv5 network makes full use of the CSP structure. The principle is as follows: Before the feature maps are sent to a series of residual modules, we extract half of the channels as the input of Branch 1, which we directly send to the output of the CSP structure. The rest of branch 2 performs intensive convolution operation, and finally performs Concat operation on the output of the two branches and merges them into one layer. The CSP structure can also be easily combined with other networks. A cross-stage connection is used in Branch 1, and Branch 2 adopts the original method of the network. The structure of CSPResNet and ResNet adopted in this paper is shown in Figure 3. The parameter n represents the number of cascaded modules.

There are two advantages by increasing the learning function of the cross-stage partial branches:

(1) The problem of gradient disappearance caused by multiple residual structures is avoided. Multi-round convolution may cause the problem of gradient disappearance in the backpropagation process of the network. The parameters are connected to the output terminal by directly drawing a branch in the CSP structure so that part of the information transmission process avoids multi-round convolution to alleviate the gradient disappearance.
(2) The number of network calculations is balanced. In addition to the disappearance of gradients, multiple convolutions will also bring a huge amount of calculation. However, when the CSP residual module performs dense convolution, only half of the channels are used, and the other half is directly connected to the output end so the part that participates in multi-round convolution calculations is less compared with the classical residual module. Nearly half of the calculation amount can be reduced in the end.

**Figure 4:**
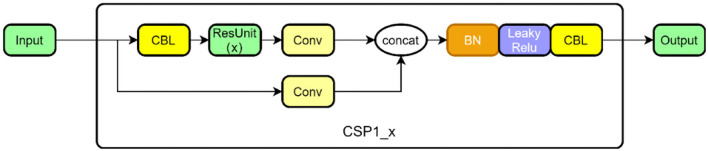
CSP1 structure in backbone.

**Figure 5:**
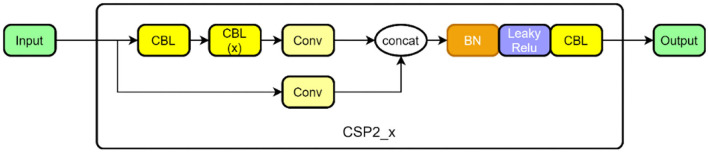
CSP2 structure in the feature analysis layer.

The CSP structure is combined with the residual network, and there are two CSP structures in Figure 4. The former is designed to be applied in the feature extraction part of the backbone network in the first half of the network, and the latter is designed for the feature analysis part, which is at the end of the network. Among them, the convolution module is an important part of the CSP structure, including the convolution layer + the BN (batch normalization) layer + the activation layer, which extracts information through the cascade. In the backbone network, the dense convolution part of the CSP structure includes a convolution module, and then features are extracted through a series of residual modules, and the other branch is merged with the dense convolution branch after a layer of convolution (Concat). At the end of the network, the dense convolution of the CSP structure does not apply to the residual module but makes use of a multi-layer cascaded convolution module instead. The other branch is the same as in the backbone network. After passing through a convolution layer, it is combined with the dense convolution branch. The structure in the backbone network is shown in Figure 4 as CSP 1, and the structure in the feature analysis network is shown in Figure 5 as CSP 2.

The YOLOv5 algorithm has achieved good results on the public dataset, but there is a certain difference between object detection of boats on the water and object detection in the public dataset. The anchor frame adaptive calculation method included in the original YOLOv5 does not match the water surface dataset enough. To solve this problem, we improved the initial value of the anchor frame to adapt to the surface object.

The difference between the shape and size of USV and the object in the public dataset is mainly reflected in the aspect ratio. The shape of the boat is mostly designed as a long rectangle, and the highest points of the boat, such as masts and antennas, are generally much smaller than the boat. Therefore, the aspect ratio of USV on the image is very large, and there is a big difference compared with the public dataset. To make the object detection algorithm fit the real frame faster, in this article, we analyzed the distribution of the width and height ratios of the boat.

The aspect ratio of the object is only a one-dimensional vector, so the fast convergence K-Means algorithm is used for clustering, and the dense value area of the aspect ratio is obtained. The anchor box of the algorithm in YOLOv5 has 3 initial values, so the cluster center is set to the results of 2.3, 3.5, and 4.8. Theoretically speaking, the aspect ratio data of the USV object are distributed around these three-aspect ratios. However, in experiments, it is found that if the anchor frame is set only to consider the cluster center of the target aspect ratio distribution, it will reduce the coverage of the three anchor frames, and it is difficult to identify objects with too large and too small aspect ratios. Additionally, if the aspect ratio of the anchor frame is not set to a value of 1:1, the accuracy of the algorithm will be greatly reduced. So, the aspect ratio is set to 1, 2.3, and 4.1. Finally, on the surface USV dataset, the accuracy is improved by 2.3% compared with the original algorithm.

We also adjusted the activation function of YOLOv5, replacing SiLu with Leaky ReLu. The difference is that, when the input is negative, the output is not all 0. Suppose the input of the activation layer is x, then the output y of Leaky Relu can be expressed as:


(1) $$y=\left\{ \begin{array}{l} x\text{ if }x\geq 0\\ ax\text{ if }x<0 \end{array}\right.$$

In this algorithm, a is 0.5. For the negative semi-axis, the input of different x will still have an impact on the network, which is smaller than the positive semi-axis but can optimize the transmission of the gradient. When the input of the SiLu function used by the original network is < -1, the output will become 0, which will invalidate the neuron and affect the recognition of surface ships.

### Object Tracking for USV

To ensure the real-time performance of the algorithm, the tracking part adopts the KCF algorithm, which has a great advantage in speed. At the same time, considering the characteristics of large changes in the scale of the water surface target, a multi-scale adaptive module is added.

The algorithm uses the gradient histogram feature (FHOG) to describe the image and uses the ridge regression model to optimize the weight coefficient w of the filter. The objective function of ridge regression is:


(2) $$\begin{array}{r} \underset{\text{w}}{\min}\underset{i}{\sum }{\left(f\left({\text{x}}_{i}\right)-y_{i}\right)}^{2}+\lambda ||\text{w}|{|}^{2} \end{array}$$

functional of training is to find a function *f*(*z*) = *w*^*T*^*z* that minimizes the squared error over samples *x*_*i*_ and their regression targets *y*_*i*_. The λ is a regularization parameter that controls overfitting. The minimizer has a closed form, which is given by


(3) $$\begin{array}{r} \text{w}={\left(X^{H}X+\lambda \right)}^{-1}X^{H}Y \end{array}$$

where X is the input feature matrix, Y is the input label, and *X*^*H*^ is the Hermitian transpose. Equation (3) is the vector form of the filter parameter solution.

The original target sample is cyclically shifted, and then a large number of training samples can be obtained, and data enhancement can be realized. According to the diagonalization of the circulant matrix, the operation in the time domain can be transformed into the frequency domain:


(4) $$\begin{array}{r} X=Fdiag\left(\hat{\text{x}}\right)F^{H} \end{array}$$

where F is a constant matrix that does not depend on x, and X=F(x).

To simplify the calculation, the features obtained by linear space ridge regression are mapped to high-dimensional space through the kernel function. Through the mapping function, the filter becomes:


(5) $$\begin{array}{r} f\left({\text{x}}_{i}\right)={\text{w}}^{T}\varphi \left({\text{x}}_{i}\right) \end{array}$$

Since the filter parameters can be expressed using a linear combination of input eigenvectors, we suppose $w=\underset{i}{\sum }\alpha_{i}\varphi \left(x_{i}\right)$ so that the solution of *w* becomes the solution of α. The kernel function can be constructed, and the solution of the ridge regression based on the kernel function and Fourier diagonalization can be obtained:


(6) $$\begin{array}{r} \alpha ={\left(K+\lambda I\right)}^{-1}\text{y} \end{array}$$

where K is the kernel matrix and α is the vector of coefficients α_*i*_, which represent the solution in the dual space.

The response of the test sample is as follows:


(7) $$\begin{array}{r} f\left(z_{j}\right)=â\phi \left(X\right)\phi \left(z_{j}\right) \end{array}$$

**Figure 6:**
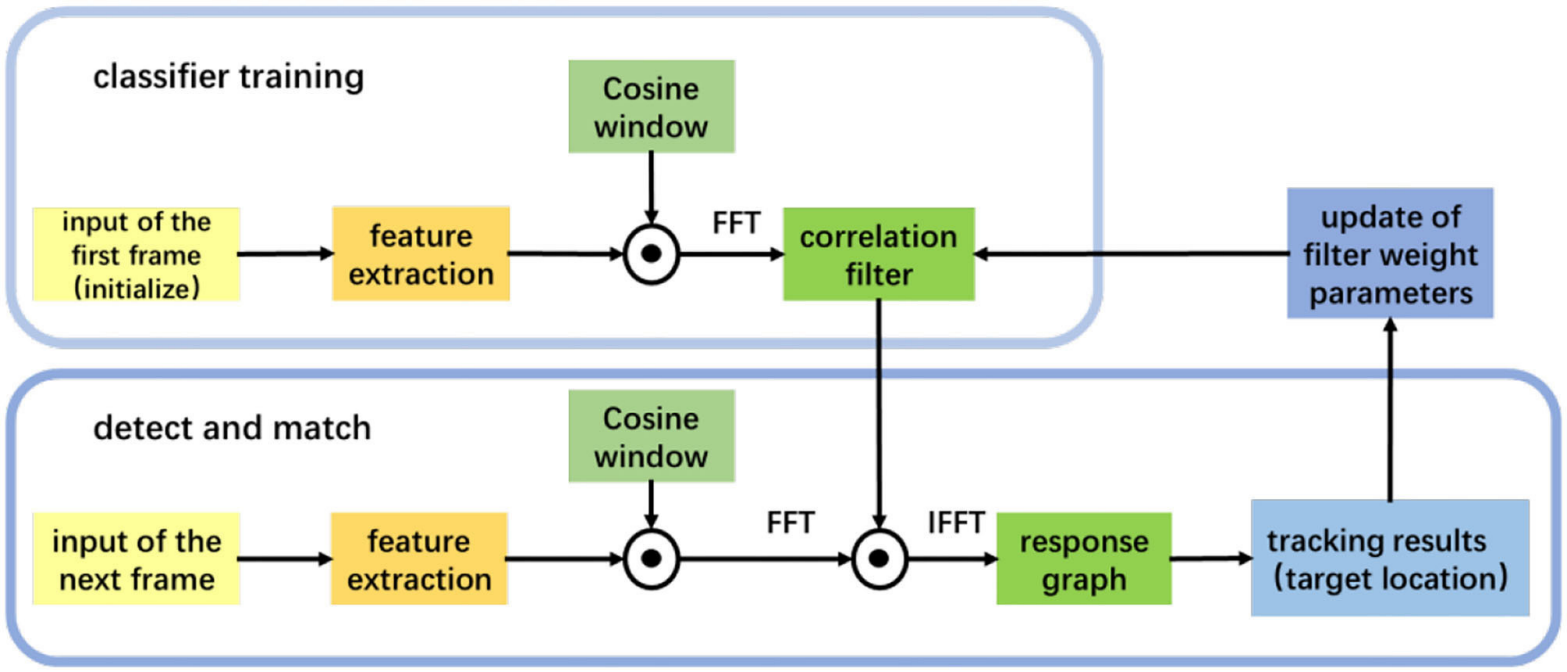
An overall framework of the tracking algorithm.

The tracking algorithm framework is shown in Figure 6. Firstly, we extract the HOG feature from the initial image, train the filter, and obtain the filter template. In the next input video, we extract the feature and perform correlation calculation with the filter template to obtain the feature response map. The coordinates are the object position.

The water surface environment has a wide field of vision and the scale of the target changes during the movement. Therefore, based on the original template, two additional templates with different scales and the same center point coordinates are calculated, and the response is finally obtained, and the strongest response is obtained after comparison:


(8) $$\begin{array}{r} R=\max\left(R_{0},R_{+1},R_{-1}\right), \end{array}$$

where *R*_0_, *R*_+1_, and *R*_−1_ are the response of three different templates, and R is the final response obtained.

The most responsive template is used as the output scale, which realizes multi-scale adaptation, has a small amount of calculation, and is highly efficient and feasible.

### Limitations of Detection and Tracking

Both object detection and object tracking have certain limitations. Object detection extracts single-frame image features, which has the advantages of higher accuracy and robustness, but it lacks correlation of time information between different frames of the video image, resulting in lower speed and missed inspections. The object tracking algorithm is realized by extracting the correlation characteristics of continuous frame images and training the filter. The calculation amount is small, and it has the advantage of fast running speed, but it is easy to accumulate errors and cause target drift.

**Figure 7:**
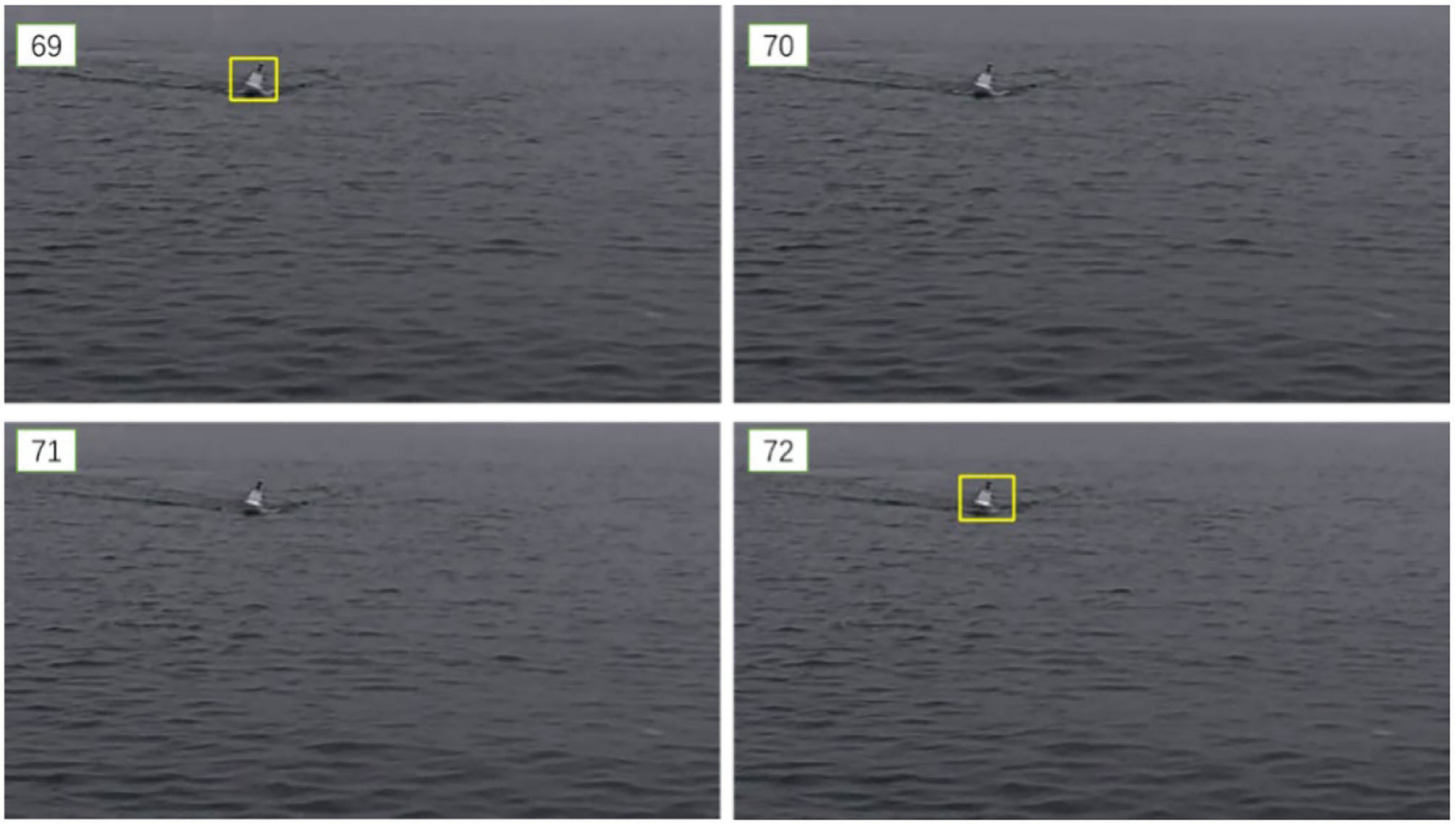
Missed detection circumstances.

In the video displayed in Figure 7, an obvious problem of missed detection can be seen. Among the 69–72 frames of images, only the first and last frames have the target detected. The shape of the unmanned boat in these frames hardly changes, but the rate of missed detection can be as high as 50% in the four frames of the intercepted fragment. Such a missed detection rate obviously cannot support the task of autonomous obstacle avoidance and unmanned boat confrontation.

There are three main reasons for the missed detection situation:

1. The object detection algorithm does not use the relationship between video frames. The purpose of the object detection algorithm is to classify and locate the target appearing in a single image, so only the features of one frame of the image are extracted and analyzed. The object generally exists in multiple consecutive frames, and the detection of adjacent frame image features can also be used as a reference basis for detection and recognition. This part of the feature is not used in the target detection process. Therefore, even if the object is detected in adjacent frames, it is difficult to use the information in different video frames.
2. The scale of the boat object in the image is relatively small. The accuracy of the object detection algorithm in the public data set can reach about 80%, which is much higher than in Figure 7. This is because, under the background of USV confrontation, it is necessary to detect and track the USV when it is far away. In this case, the size of the unmanned boat is small, the resolution is insufficient, and it is difficult for the algorithm to extract sufficient features, which would be used for judgment, thereby affecting the recognition effect.
3. The image quality under the water surface environment is limited. Most of the objects in the public data set are clear, while the objects on the water are more susceptible to light and waves, making the image quality poor. This impact on image quality is difficult for the human eye to capture, but these small differences result in the different outputs of adjacent frames. The poor image quality further exacerbates the missed detection problem.

**Figure 8:**
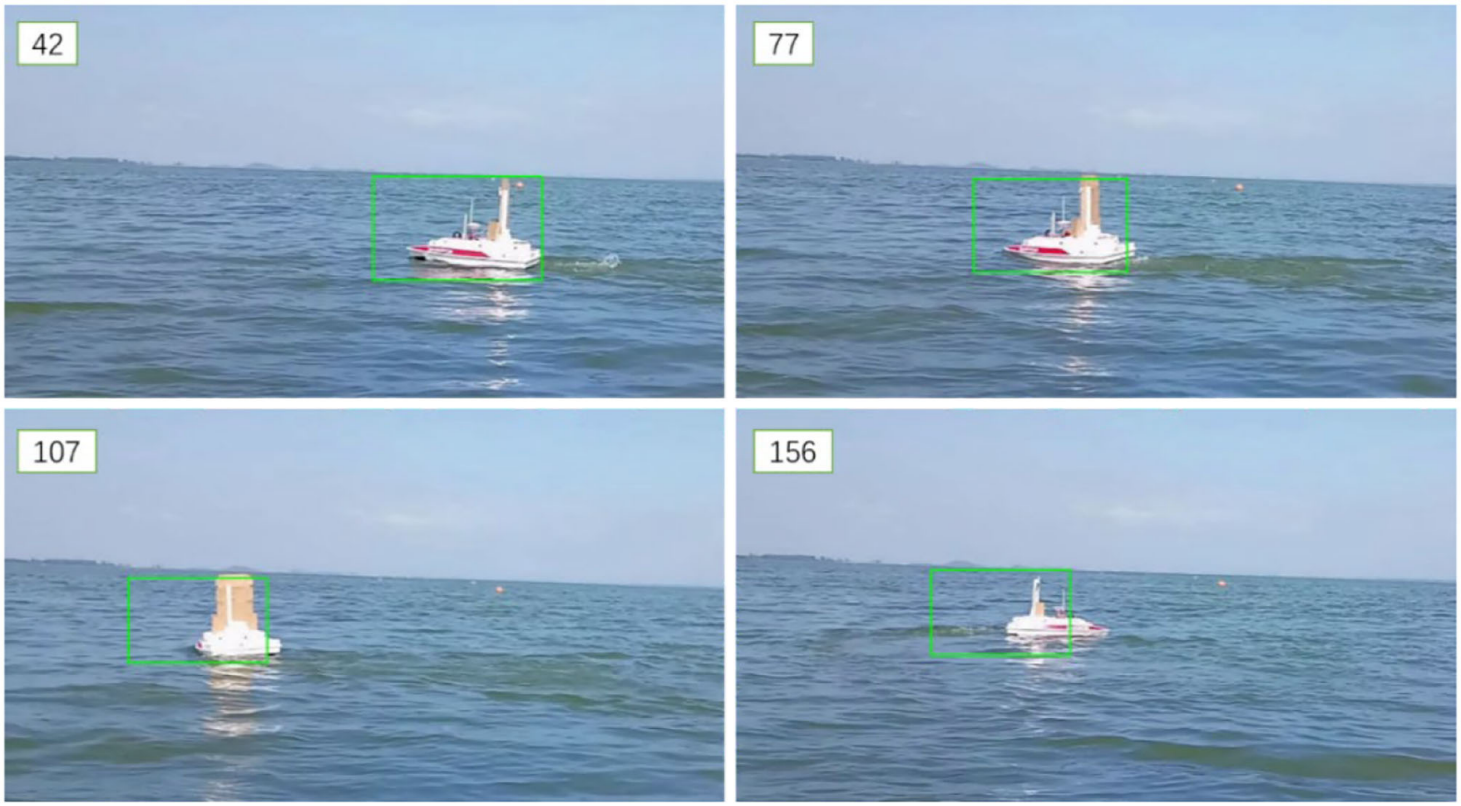
The tracking drift problem.

The object tracking algorithm has the problem of tracking drift. As shown in Figure 8, the frame number is marked in the upper left corner.

There are two reasons for drift problems in the object tracking algorithm:

1. The template tracking algorithm is difficult to keep up with when the object shape changes significantly. When the object is rapidly deformed, the characteristics of the object will also change rapidly. The template update of the tracking algorithm is determined by the current frame and multiple frames in the past. Although the current frame has learned the feature change, the previous frame still retains the feature before the deformation, and, finally, under the weighting of the current frame and the past frame by the learning rate parameter, the algorithm tends to use the features in the past frame to match the object position so that the tracking frame cannot be enlarged or zoomed out with the deformation of the object.
2. A large amount of similar background noise in the water surface environment. The existence of background noise makes it impossible to increase the learning rate parameter to solve the problem of the drift because when the learning rate parameter is increasing, it means that the information of the current frame will be more reflected in the template, and noise will inevitably be introduced into the tracking. If background noise is learned in the template of the current frame, it will have a greater response to the background in the next frame, and the background noise on the water surface is relatively similar, so the response of the template may quickly deviate from the target area, causing a greater degree of drift. The error caused by drift will increase with the accumulation of time. There is only a slight drift in the 77th frame, while the drift in the 156th frame is very visible.

### Fusion Module

**Figure 9:**
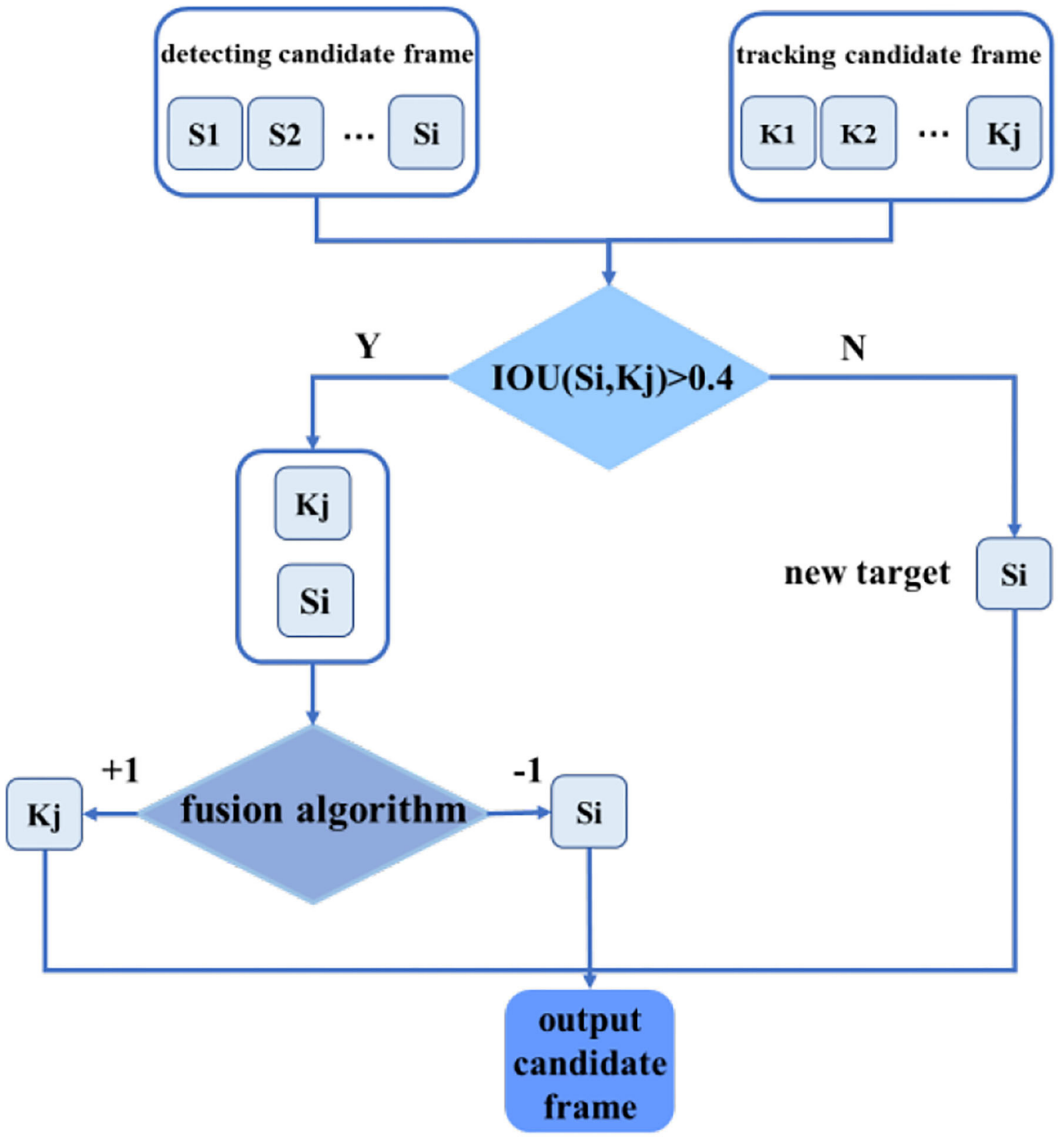
A candidate frame selection strategy flow chart.

In response to the above problems, time feature information and spatial feature information are integrated, and the advantages of both are used to improve the detection and tracking effect of USV objects. Firstly, the detection algorithm is used to determine the position of the object in the first frame of the image, and then the position is used as the input information of the tracking algorithm, and the object is tracked for 40 frames to avoid the problem of missed detection. Due to the good real-time performance of the tracking algorithm, the running speed of the algorithm can also be improved. After 40 frames of tracking, a re-detection mechanism needs to be run to ensure the accuracy of continuous video tracking, and the newly emerging objects are input into the tracking algorithm for initialization. The setting of parameters is based on rules of thumb. In our algorithm, the frequency of detection would influence both accuracy and computing speed. We tested a series of values and found that the algorithm can perform better with parameters set as 40 frames. The fusion block diagram is shown in Figure 9.

Firstly, we calculated the degree of coincidence between the detection candidate frame and the tracking candidate frame and judge whether the detection result and the tracking result are the same targets. In this article, we used the intersection-union ratio (IOU) as the criterion for judging the degree of coincidence. IOU is a standard for measuring the accuracy of detecting corresponding objects in a specific data set. IOU is a simple measurement standard. As long as it is a task to obtain abound in the output, IOU can be used to measure. IOU means the ratio of the intersection of the two candidate frames to the union. The calculation method is as follows:


(9) $$\begin{array}{r} \text{IOU}=\frac{S_{i}⋂K_{j}}{S_{i}⋃K_{j}} \end{array}$$

If ∀*K*_*j*_, *IOU*(*S*_*i*_, *K*_*j*_) <0.4, it will be regarded as a new target and output, participating in the initialization of the tracking algorithm. If ∃*K*_*j*_, *IOU*(*S*_*i*_, *K*_*j*_)>0.4, it is considered that the detection frame and the tracking frame have detected the same target. At this time, the confidence level of the output frame of the detection algorithm conf(*S*_*i*_)is compared with the normalized response of the output frame of the tracking algorithm. However, it is difficult to directly compare the value of the two different algorithms' confidence. Therefore, based on the above detection and tracking, the additional features of the target are extracted, and the support vector machine is used for fusion. Since the confidence of the detection algorithm is obtained through multiple rounds of convolution, it is robust to the different foreground and background inputs, and the confidence of the output is relatively reliable; However, the normalized response output of the target tracking algorithm has a large fluctuation in value with the difference of the input. In order to improve the robustness of the output response of the tracking algorithm, the response mean value avg(*K*_*j*_) and the tracking frame scale of the 3 × 3 areas centered on the peak of the response graph are additionally extracted, and the detection confidence and tracking response are input to the support vector machine for classification, and the output −1 means that the result of the detection algorithm is better than that of the tracking, and the output +1 means that the value of the tracking frame is more accurate. Using a more accurate frame as the initial value of the tracking algorithm can reduce the introduction of background noise, thereby reducing the possibility of tracking frame drift.

## Results

**Figure 10:**
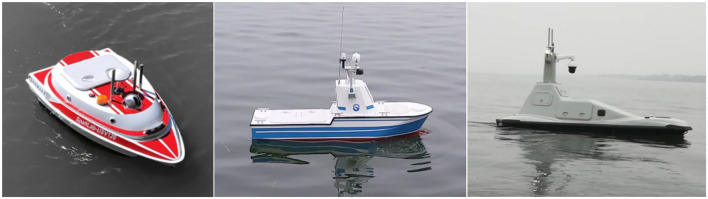
Unmanned surface vehicles for the experiment.

The experiment uses three different sizes of unmanned boats: USV-120, USV-180, and USV-320 independently developed by the team as the tested objects. The lengths of the three boats are 1.2, 1.8, and 3.2 m, respectively, as shown in Figure 10.

In this paper, 1,550 photos are collected as a picture set; 80% of which are used for training, and 20% are used for testing detection algorithms. The learning rate was set to 0.001, and 30,000 pieces of training were performed on NVIDIA GeForce GTX 1080TI, and the average accuracy on the test set was finally 93.8%. We select 3 pieces of video to analyze the effect of the fusion algorithm. In Video 1, the object is 30 m away from the camera to test the effect of the scale change of the USV. Video 2 tests the impact of the viewing angle change on the detection and tracking, and the object is 60–70 m away from the camera. Video 3 is the detection effect of the USV at a long distance. The distance is more than 100 meters, and the object scale is <0.5% of the original image. The evaluation index used on the video set is the success rate. When the overlap rate of the output of the algorithm and the real frame of the video exceeds a certain threshold, it is regarded as a successful recognition. The ratio of the number of frames successfully recognized to the total number of video frames is the success rate. The algorithm test platform is the NVIDIA Jetson TX2 board, which contains CPU and GPU, and its power consumption is only 15W, which is convenient for deployment on unmanned systems.

**Figure 11:**
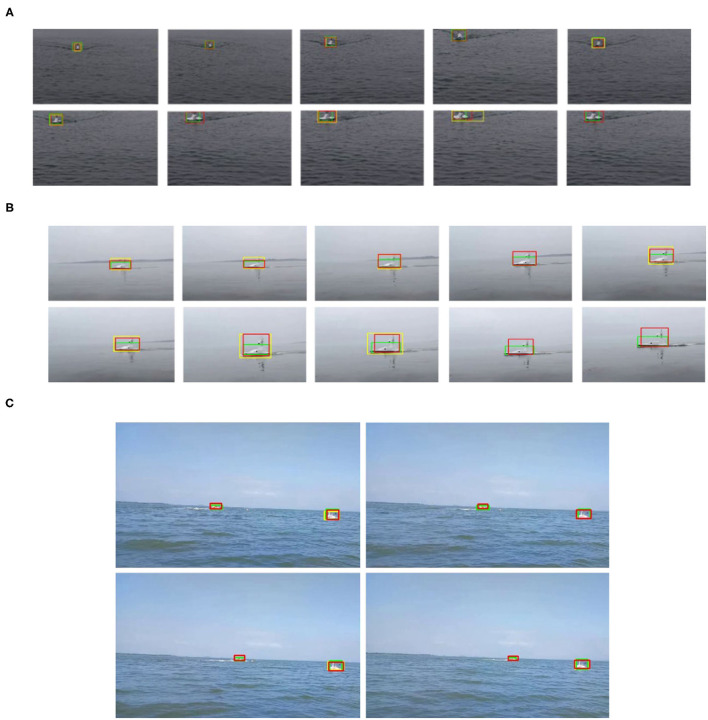
Comparison of detection and tracking results of three fusion algorithms in Videos 1~3 **(A–C)**. Red bounding box: fusion algorithm, yellow bounding box: YOLOv5, green bounding box: KCF.

The detection and tracking effect of the USV target is shown in Figure 11, where the yellow box is the detection algorithm, the green box is the tracking algorithm, and the red box is the fusion algorithm. Visibly, the yellow detection frame is not marked in some frames, indicating that the detection algorithm has a certain degree of missed detection, and the result of the green tracking algorithm often cannot accurately frame the object, which is caused by the drift of the tracking frame. The overall effect of the fusion algorithm's output red frame is better, which solves the problems of missed detection and drift problems to a certain extent.

**Figure 12:**
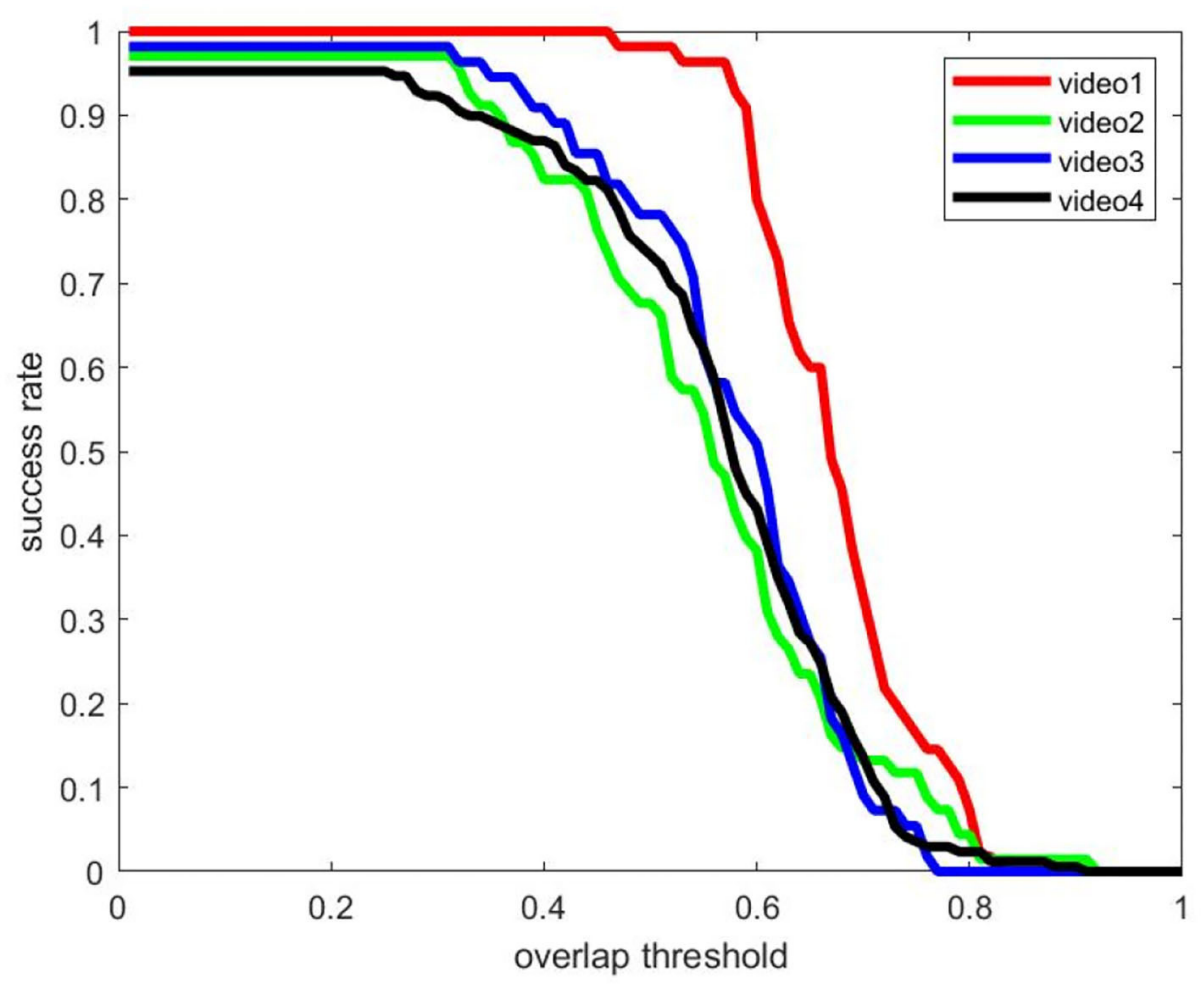
The success rate of the test dataset.

The quantitative analysis of the algorithm is shown in Table 1; Figure 12. When the object overlap ratio (IOU) threshold is set to 0.5, the average coincidence rate of the fusion algorithm on the video set is 81.3%, while the average success rate of a single detection algorithm is 59.7%, and the average success rate of a single tracking algorithm is 69.5%. The success rate of the video set is numerically lower than that of the picture set. This is because the image quality in the actual collected video is not often ideal; the object is not clear, and the distance is too far. But the video set is more compatible with the picture set when applied on specific scenes.

**Table 1:** An algorithm processing result on the video set (IOU@.5).

**Video number**	**Environmental impact factors**	**Success rate for detection only**	**Success rate for tracking only**	**Success rate for fusion algorithm**	**Velocity (FPS) for fusion algorithm**
1	Scale change	0.927	0.872	0.982	40
2	Scale change	0.426	0.529	0.676	25
3	Small target	0.436	0.709	0.782	38
4	Small target	0.473	0.84	0.734	38

## Discussion

**Figure 13:**
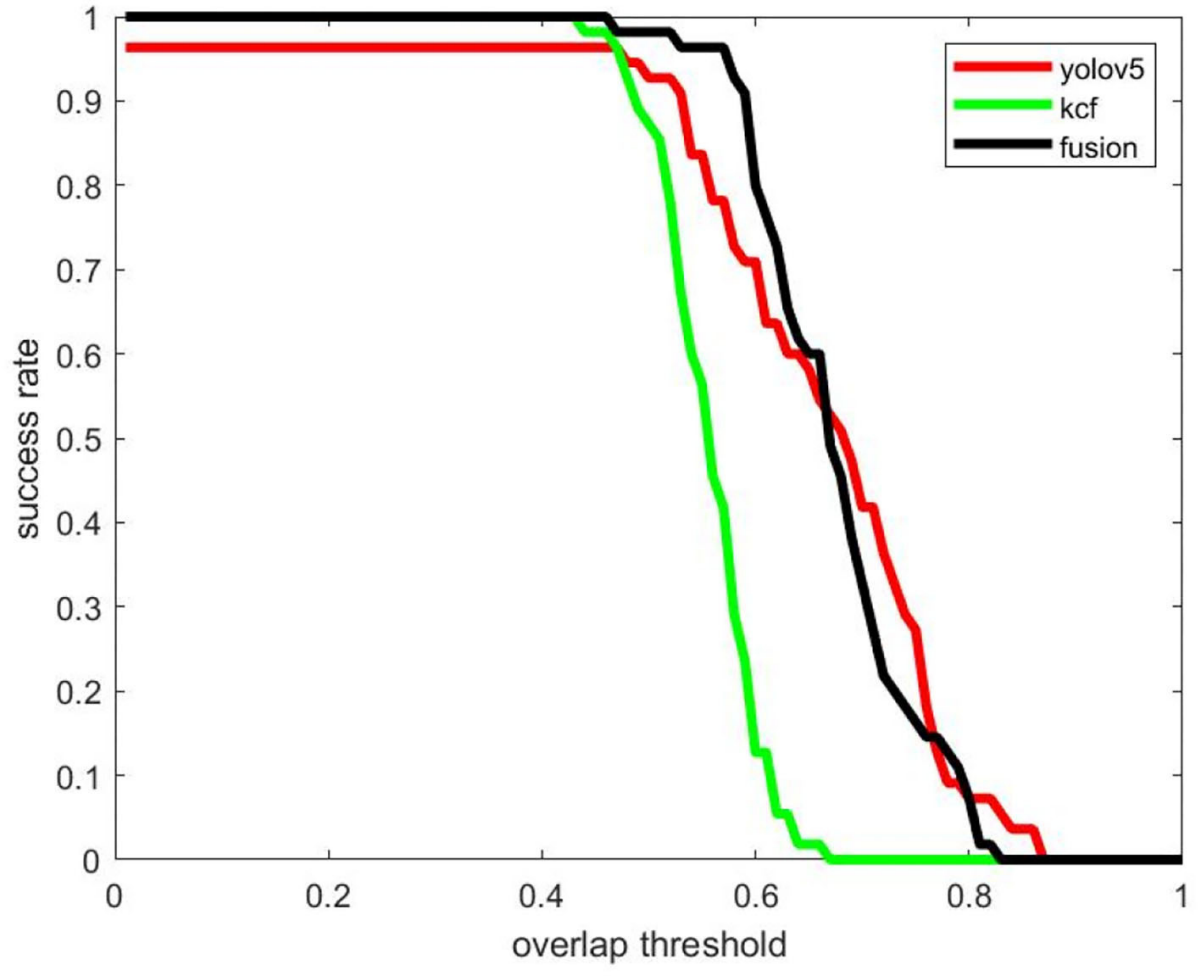
Comparison of detection and tracking success rates using KCF, Yolov5, and fusion algorithms (Video 1).

It occurs to Video 1 the problem of changing the scale from large to small, and the experimental results are shown in Figure 13. The size of the USV-320 itself in this video is larger than the USV-120, so it occupies more pixels in the image, and the extraction of target features is relatively easy. The output frame is more accurate when the object is detected on a single frame. At this moment, the detection algorithm works well. It is useful for tracking, but the problem of missed detection of some frames may also occur. The reason for the poor effect of the tracking algorithm is drifting. The drifting phenomenon of tracking can be observed in Figure 11A. The green frame of the tracking output only contains the main part of the hull in the figure, and the mast and antenna areas are not detected. This is because the tracking algorithm considers that there is more background information near the mast in the process of continuously updating the template, while the frame of the hull part contains less background, and the response is relatively high, which makes the tracking algorithm tend to use smaller frames as output. The fusion algorithm avoids missed detection by focusing on tracking and uses the detection algorithm to correct the drift of the holding frame. The highest success rate is achieved when the threshold is 0.5. Although the detection algorithm will have a small amount of missed detection, the output result is more accurate. In the case of a high overlap threshold, a single detection algorithm shows better results. However, in general, the international community does not pay attention to the success rate under excessively high coincidence. The missed detection problem that occurs in the detection has a greater impact on the perception system. Overall, the effect of fusion on the video is better.

**Figure 14:**
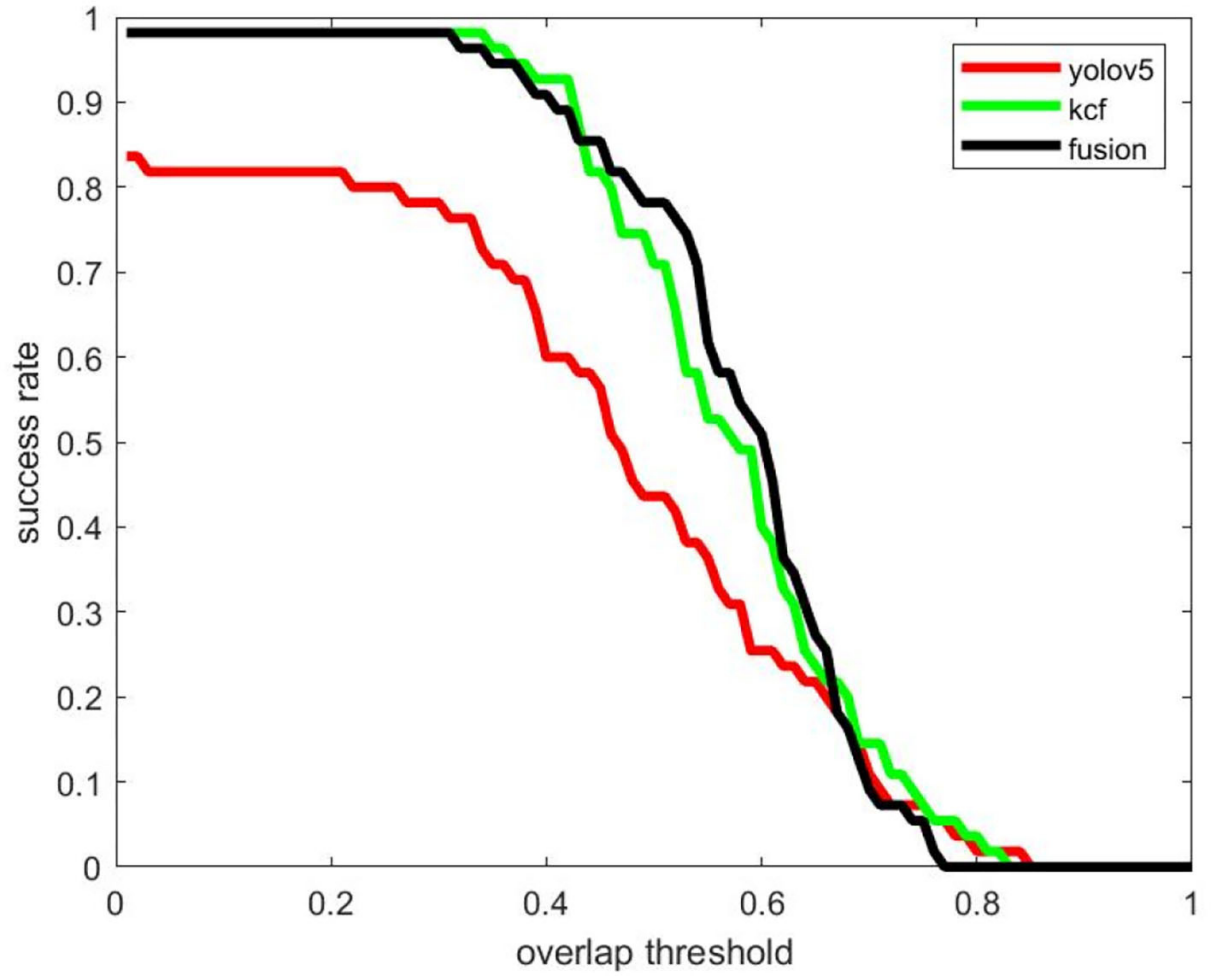
Comparison of detection and tracking success rates using KCF, Yolov5, and fusion algorithms (Video 3).

In Video 3, we mainly tested the detection and tracking effect of long-distance USV objects, and the success rate curve is shown in Figure 14. Since the measured object is far from the camera, the problem of missed detection is obvious. Even if the overlap threshold is set to 0.1, the detection algorithm still has a large number of targets difficult to detect. The KCF tracking algorithm performs better in the tracking of extremely small targets. There is almost no missed detection, and the overlap between the output frame and the real frame is also quite high. The fusion algorithm can accurately judge the results of the two algorithms, retaining the high success rate of the tracking algorithm, and, at the same time, after the detection algorithm gets better results, it can output better results through the fusion algorithm and participate in the tracking initialization. So the fusion algorithm can work better under the overlap threshold of 0.5.

The next design experiment proves the effectiveness of the fusion algorithm. This paper extracts 50 sets of detection and tracking samples in video sets 2 and 3 for the fusion part of the training algorithm and conducts actual tests on 180 samples selected in the entire video set. The number of test and training samples is in the video set. The distribution is shown in Table 2, where the detection accuracy is greater than the tracking samples accounting for about 65% of the total.

**Table 2:** The fusion algorithm test.

**Dataset**	**Video 1**	**Video 2**	**Video 3**	**Video 4**
Training	0	37	13	0
Test	47	51	82	48

Experiments show that the accuracy of the test set can reach 87.5%, indicating that the support vector machine can select a more reliable output in the fusion stage, and the input features selected by the algorithm, namely, target scale, tracking response graph partial mean value, tracking response peak. The confidence levels of target detection and object detection both play a role in the fusion process.

We also tested on Singapore maritime dataset (SMD) (Kahou et al., 2017) and multi-modal marine obstacle detection dataset 2 (Prasad et al., 2017) (MODD2), which are publicly available marine datasets for both object detection and object tracking.

**Figure 15:**
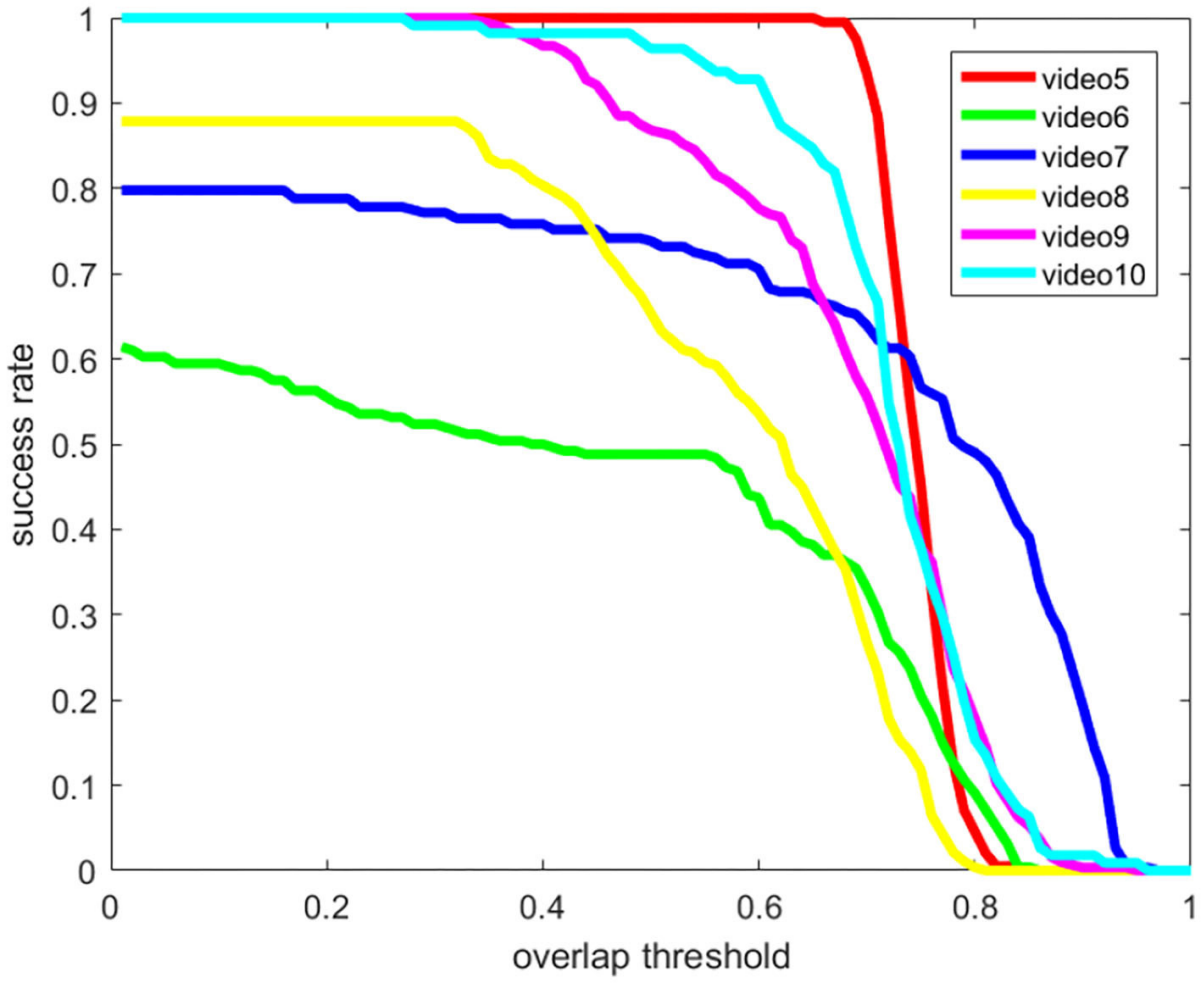
The success rate of public datasets.

**Figure 16:**
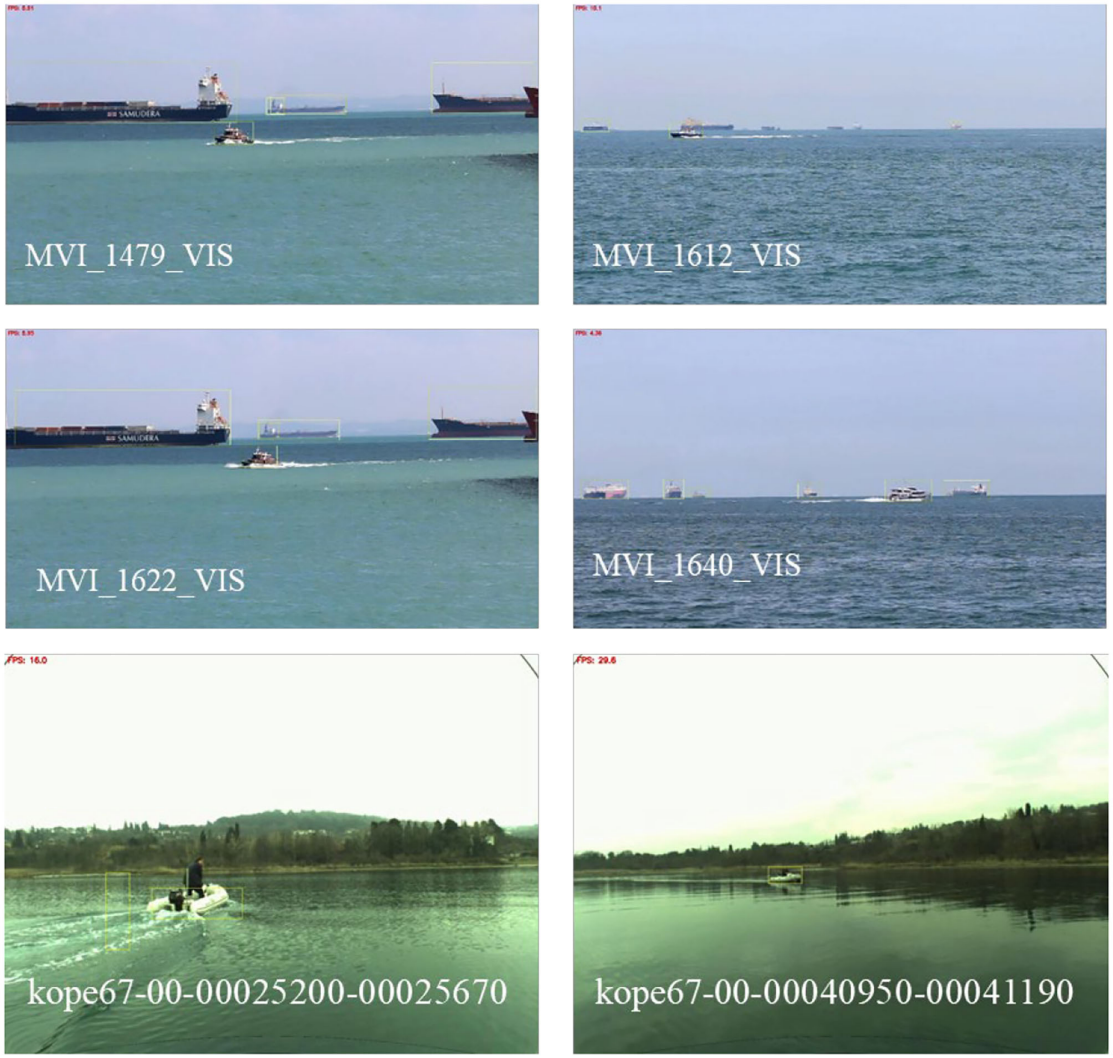
Results of public datasets.

There are 40 videos in the on-shore class of the SMD dataset, and 4 representative videos (Videos 5–8 in Figures 15, 16) are selected to verify the effectiveness of our method. Multi-modal marine obstacle detection dataset 2 (MODD2) consists of 28 video sequences of variable length, amounting to 11,675 stereo-frames at resolution of 1,278 x 958 pixels each. So, we reverted the video sequences to original videos by frame and tested 2 representative videos (Videos 9–10 in Figures 15, 16). In the background of the sea and sky, certain large ships remain relatively stationary, but we pay more attention to the moving boats for object tracking. Therefore, we extracted data of moving boats for testing. Compared with the previous experiment, the boats in these datasets are smaller in the field of view, which puts forward higher requirements for the performance of the algorithm. The overall average accuracy is relatively lower than before, but it still has a good recognition effect on small moving objects. The results are shown in Figures 15, 16 (Video 5: MVI_1479_VIS; Video 6: MVI_1612_VIS; Video 7: MVI_1622_VIS; Video 8: MVI_1640_VIS; Video 9: kope67-00-00025200-00025670; Video 10: kope67-00-00040950-00041190).

## Conclusions

To meet the needs of USV confrontation on the surface battlefield in the future, in this paper, we propose a detection and tracking fusion algorithm for USV objects. For the tracking drift problem, the deep learning detection algorithm is used to extract the single-frame image information to accurately detect the position of the target; for the missed detection problem, the relevant filter tracking is used to obtain the time information, making full use of the inter-frame correlation characteristics; finally, the fusion algorithm is designed to make the detection information and tracking information become complimented to meet the reconnaissance missions of unmanned boat targets in different scenarios. To verify the effectiveness of the system, experiments were conducted on three types of unmanned boats. The results show that the detection and tracking fusion algorithm can efficiently detect and track the unmanned boat targets in different scenarios, and the success rate exceeds that of a single detection and tracking algorithm.

## Data Availability Statement

The original contributions presented in the study are included in the article/supplementary material, further inquiries can be directed to the corresponding author.

## Author Contributions

ZZ initiated and supervised the research. XH and ZJ wrote the paper and carried out the experiments. ZL polished the paper. CQ put forward some effective suggestions for improving the structure of the paper. All authors contributed to the article and approved the submitted version.

## Funding

This work is supported by the Equipment Pre-Research Field Fund 13th Five-Year (No. 61403120109, BIT).

## Conflict of Interest

CQ was employed by China State Shipbuilding Corporation Limited. The remaining authors declare that the research was conducted in the absence of any commercial or financial relationships that could be construed as a potential conflict of interest.

## Publisher's Note

All claims expressed in this article are solely those of the authors and do not necessarily represent those of their affiliated organizations, or those of the publisher, the editors and the reviewers. Any product that may be evaluated in this article, or claim that may be made by its manufacturer, is not guaranteed or endorsed by the publisher.
